# Comparative machine learning analysis of saliva and plaque microbiomes in Kuwaitis with type 1 diabetes

**DOI:** 10.3389/fmicb.2026.1735375

**Published:** 2026-03-27

**Authors:** Hend Alqaderi, Rebecca Batorsky, George Azar, Md. Zubbair Malik, Rasheeba Nizam, Khaled Altabtbaei, Sriraman Devarajan, Rasheed Ahmad, Dominique S. Michaud, Naisi Zhao, Athanasios Zavras, Fahd Al-Mulla

**Affiliations:** 1 Tufts University School of Dental Medicine, Boston, MA, United States; 2 Dasman Diabetes Institute, Kuwait City, Kuwait; 3 Tufts Institute of Artificial Intelligence, Tufts University, Medford, MA, United States; 4 Tufts University School of Medicine, Boston, MA, United States

**Keywords:** biofilm, diabetes, microbiome, oral, plaque, saliva, type 1 diabetes

## Abstract

**Background:**

Type 1 diabetes (T1D) is associated with microbial dysbiosis. While most research has focused on the gut microbiome, limited data addresses the role of the oral microbiome in T1D. The oral and gut microbiomes overlap substantially, and the oral cavity may influence gut microbial composition. Saliva and dental plaque represent two distinct oral niches with unique microbial communities, but it remains unclear which is better associated to systemic disease states like T1D. This study compares the performance of salivary and plaque microbiomes in classifying pediatric T1D status.

**Methods:**

Paired saliva and plaque samples were collected from 46 children (23 with T1D, 23 healthy controls). Microbial DNA was extracted and sequenced targeting the 16S rRNA gene. Data were processed using QIIME 2 for taxonomic classification and centered log-ratio transformation. Alpha diversity, microbial abundance, and clustering analyses were performed to compare the oral microbiome between T1D and control groups. Random forest classifiers were used to evaluate and compare the predictive accuracy of saliva- and plaque-based models, both with and without clinical metadata.

**Results:**

Saliva samples exhibited lower alpha diversity than plaque but had significantly higher bacterial load and total microbial abundance. Saliva-based models outperformed plaque-based models, achieving a classification accuracy of 94.2% with or without clinical metadata, compared to 73.3% accuracy for plaque-based models. ROC curve analysis further supported this difference, with saliva models reaching an AUC of approximately 0.94, versus 0.75 for plaque, indicating superior discriminative performance. UMAP clustering revealed more distinct separation of T1D and control groups in salivary profiles than in plaque. Feature importance analysis identified both unique and shared taxa predictive of T1D in each niche. Incorporating clinical and demographic metadata did not enhance model performance, underscoring the robustness and predictive strength of microbiome data alone.

**Conclusion:**

The salivary microbiome is a more effective biospecimen than dental plaque for characterizing T1D-associated microbial profiles in children. It offers superior classification accuracy and greater sensitivity in distinguishing T1D status, supporting saliva’s potential as a non-invasive, scalable medium for future microbiome-based monitoring.

## Introduction

According to data from 45% of countries, the global prevalence of T1D in children was estimated at 1,110,100 cases in 2019 (Patterson et al., 2019). The etiology of T1D is multifactorial, involving genetic susceptibility, lifestyle factors, viral infections, and gut microbiota dysbiosis (Shilo et al., 2022). Emerging evidence suggests that the human microbiome plays a crucial role in biological processes such as immunity, inflammation, and glucose metabolism (Liu et al., 2021), all of which are relevant to the pathogenesis of T1D. This growing understanding has led some researchers to refer to the human microbiome as the “new organ” of the body (Liu et al., 2021).

The oral cavity is the second-largest microbial habitat in the human body, containing about 26% of the body’s bacteria, compared to 29% in the gut (Peterson et al., 2009). While the oral and gut microbiomes have distinct signatures (Park et al., 2021), 45% of the gut microbiome overlap with the oral microbiome, influencing gut bacteria through enteral and hematogenous routes (Park et al., 2021; Maki et al., 2021; Segata et al., 2012). While the role of the gut microbiome in glycemic control and systemic inflammation is well-documented, (Abuqwider et al., 2023; Bell et al., 2022; Xu et al., 2022; Yuan et al., 2022), the impact of the oral microbiome on T1D outcomes remains largely underexplored. Our study addresses this critical knowledge gap by identifying stable oral microbiome species associated with T1D.

Emerging evidence showed that oral microbiome in individuals with T1D differs from that of healthy individuals (Chakraborty and Ghosh, 2019; Moskovitz et al., 2021; Pachoński et al., 2021; Siudikiene et al., 2006). Further, it was found that new-onset T1D leads to oral microbiota dysbiosis, characterized by an increased presence of opportunistic pathogens, which could be partially reversed through improved glycemic control (Yuan et al., 2022).

The oral microbiome is typically studied using two primary sites: dental plaque and saliva. Previous research has consistently reported distinct microbiome profiles between these two sites (Lee et al., 2021; Mason et al., 2013; Segata et al., 2012; Wade, 2021). Both plaque and saliva microbiomes have been associated with diabetes-related outcomes (Crusell et al., 2020; Demmer et al., 2019; Shi et al., 2020; Tam et al., 2018). However, the relative utility of these microbiome sources in predicting outcomes related to T1D remains unclear.

This study aims to determine which oral microbiome site, saliva or plaque, offers greater sensitivity and classification accuracy for T1D. We also assessed key characteristics, such as microbial diversity and abundance, in both sources to identify features that may inform future testing and targeted interventions. Specifically, we compared the predictive accuracy of machine learning models using saliva and plaque microbiome data to distinguish the most effective sample type for classifying T1D status.

## Methods

Ethical approval for this study was obtained from the Kuwait Ministry of Health, Tufts University, and Dasman Diabetes Institute in Kuwait. Data were collected from 46 children aged 10–21 years, including 23 diagnosed with T1D and 23 non-diabetic controls. The research team included a coordinator, licensed dentists, and a phlebotomist. Participants were recruited from diabetes centers, hospitals, and public schools across Kuwait. Children were eligible for inclusion if they: (1) were between 10 and 21 years old, (2) were of any gender or nationality, and (3) provided informed consent/assent. Children with autoimmune diseases or cancer were excluded. Eligible participants were approached in person and given detailed explanations of all study components. Upon receiving informed consent from participants or their guardians, appointments were scheduled with matched controls of similar age.

### Saliva collection

Pre-labeled 15 mL sterile plastic tubes were used for saliva collection. Participants were instructed to rinse their mouths with a sip of water, swallow, and then passively drool into the collection tube until reaching the 4 mL mark. The tubes were sealed, disinfected with alcohol, and stored in a cooler with ice for transport. Samples were processed the same day in the laboratory, following a standardized protocol. Each sample was divided into four 1 mL aliquots and stored at −80 °C until analysis.

### Plaque collection

Plaque samples were collected prior to oral examinations to avoid disruption. Using sterile toothpicks, plaque was collected from the mesio-buccal surfaces of the six Ramfjord teeth (#16, #21, #24, #36, #41, #44) and transferred into 1.5 mL tubes containing RNALater (Invitrogen, MA, USA). Each sample was labeled, kept on ice, and stored at −80 °C until further use.

### Periodontal examination

A full periodontal assessment was performed around the Ramfjord teeth (Rams et al., 1993), progressing systematically from sextant 1 to 6. Pocket depths were measured using a calibrated periodontal probe at six sites per tooth: mesiobuccal, midbuccal, distobuccal, mesiolingual, midlingual, and distolingual. Additional indicators, including bleeding on probing and the presence of plaque or calculus, were recorded electronically. Dentists underwent calibration prior to data collection to ensure inter-examiner consistency.

### Dental caries examination

Caries assessments were performed using a dental mirror and explorer under standardized lighting. The World Health Organization (WHO) index was used to record the number of decayed, missing, and filled teeth (DMFT). All dentists were calibrated in advance to ensure standardized scoring across the cohort.

Waist circumference: was measured with a paper tape at the midpoint between the rib cage and iliac crest, during minimal respiration, to the nearest 0.1 cm.

### Questionnaire data

Participants responded to a questionnaire on iPads using RedCap software. This questionnaire included validated questions on diet pattern using the Growing Up Today Study (GUTS) questionnaire (Rockett et al., 2007), sleep behavior (Nascimento-Ferreira et al., 2016), smoking (Wu et al., 2023), physical activities, oral health and medical history.

### DNA extraction and 16S rRNA gene sequencing

Microbial DNA was isolated from saliva and plaque samples using the PureLink™ Microbiome DNA Purification Kit (Thermofisher, USA) and quantified using a Qubit 4 fluorometer (Thermofisher, USA) in accordance with the manufacturer’s instructions. The amount of DNA used for each library preparation is 5 ng/μL of 2.5 μL of purified DNA from both saliva and plaque samples. DNA was amplified using gene-specific primers with overhang adapters attached, 16S Forward Primer: 5′ TCGTCGGCAGCGT CAGATGTGTATAAGA GACAGCCTACGGGNGGCWGCAG and 16S Reverse. Primer: 5′GTCTCGTGGGCTCGGAGATGTGTA TAAGAGACAGGACTA CHVGGG TATCTAATCC, targeting the bacterial 16S rRNA V3 and V4 regions. The PCR reaction was carried out using KAPA HiFi HotStart Ready Mix PCR mix following manufacturer’s recommendation. The resulting PCR amplicons (~550 bp) were confirmed on a Bioanalyzer using Agilent DNA 1000 chip (Agilent, USA) and purified using AMPure XP beads (Agilent, USA). Dual indexing reaction was performed using the Nextera XT Index Kit (Illumina Inc., USA) following the manufacturer’s recommendations. Purified libraries were multiplexed; 4nmole of individual libraries prepared from each sample were pooled, denatured, and further diluted to a final concentration of 6 p.m. Prepared libraries were combined with 5% of denatured Phix control spike-in and the sequencing reaction was performed in MiSeq platform (Illumina Inc., USA) using MiSeq reagent kit V3 (600 cycle) kit, producing 2 × 300-bp paired-end reads.

### Computational pre-processing of microbiome data

Raw sequencing reads had primers trimmed using Cutadapt (Martin, 2011) within the QIIME 2 environment. Amplicon sequence variants (ASVs) were generated by denoising the data with the dada2 denoise-single command in QIIME 2, applying parameters of –p-trim-left 0 and –p-trunc-len 215 to ensure optimal data quality. Abundance filtering was used to eliminate spurious ASVs (Wang et al., 2018).

### Phylogenetic and taxonomic analysis

A phylogenetic tree of the ASVs was constructed using QIIME 2 commands, which include alignment with MAFFT, masking with alignment mask, phylogenetic tree construction with FastTree, and midpoint rooting of the tree to compute weighted UniFrac metrics. Taxonomic classification was performed using the q2-feature-classifier plugin in QIIME 2, referencing the SILVA database (release 132) (Quast et al., 2012).

### Statistical analysis

Statistical Analysis was performed on paired saliva and plaque samples that were collected from 46 subjects. The average sequencing depth per saliva sample was 349,200 reads (SD = 119,207), while plaque samples averaged 158,245 reads (SD = 34,784). Taxonomic classification at the species level identified 281 species in saliva and 265 species in plaque, with 231 species shared between the two sample types. After quality filtering and prevalence thresholds were applied, 194 species in saliva and 207 species in plaque were retained for downstream analysis.

To account for the compositional nature of microbiome data, centered log-ratio (CLR) transformation was applied, following Aitchison’s methodology (Aitchison, 1982). Alpha diversity was assessed using the Shannon index and compositional analyses were conducted using the Phyloseq R package. Dimensionality reduction for visualization of microbial community structure was performed using Uniform Manifold Approximation and Projection (UMAP) via the UMAP R library.

### Machine learning analysis

Supervised machine learning was performed using random forest classifiers implemented via the tidymodels framework in R, utilizing the ranger engine, to predict binary T1D status (T1D vs. healthy control). The dataset was randomly partitioned 10 times into training (75%) and testing (25%) sets using different random seeds. Model tuning was conducted on the training data using 3-fold cross-validation, testing five combinations of the *min_n* and *mtry* hyperparameters. Separate models were trained on species-level microbiome data from saliva and plaque samples, both with and without the inclusion of clinical and demographic metadata. For each run, microbial species were ranked by permutation-based feature importance, and scores were aggregated across all 10 runs. For each species, we recorded: (1) total summed importance, (2) the number of runs in which it appeared among the top 50 features, and (3) its average importance score. Each species was classified as predictive in both saliva and plaque (“shared”) or uniquely predictive in one sample type (“saliva only” or “plaque only”).

Models were also evaluated both with and without clinical metadata to assess whether metadata enhanced predictive power. Clinical variables included demographic characteristics (gender, parental education), medical history, lifestyle behaviors (dietary habits, sleep, oral hygiene, physical activity), and clinical dental metrics (DMFT scores, presence of plaque, and probing depth). Anthropometric and physiological measures (blood pressure) were also included. Model performance was assessed on the test sets using classification accuracy. Feature selection was based on species-level importance values summed over all runs, allowing for the identification of consistently predictive microbial taxa.

## Results

### Clinical and demographic profiles of participants

The demographic, clinical and oral health characteristics of the study cohort, including 23 children with T1D and 23 non-diabetic controls is summarized in Table 1. There were no statistically significant differences between groups for gender distribution (*p* = 0.76), age (*p* = 0.17), neck circumference (*p* = 0.78), waist circumference (*p* = 0.69) and systolic blood pressure (*p* = 0.76). Diastolic blood pressure showed a borderline difference, with T1D participants exhibiting slightly higher values compared to controls (74.85 ± 8.65 mmHg vs. 70.00 ± 8.02 mmHg; *p* = 0.05). Periodontal status, as assessed by probing depth, and bleeding on probing were similar between groups, with no significant differences in the number of sites with probing depth ≥4 mm (*p* = 1) or the average number of bleeding sites (*p* = 0.77). The DMFT index, reflecting cumulative dental caries experience, was also comparable between T1D and control children (6.35 ± 4.56 vs. 7.52 ± 4.97; *p* = 0.40). These results indicate that the T1D and control groups were generally well-matched in terms of demographic and oral health parameters.

**Table 1:** Demographic and clinical characteristics of study participants.

Covariate		T1D	Control	Count/overall mean	*p*-value
Gender	Male	13 (28%)	11 (24%)	24	0.76
	Female	10 (22%)	12 (26%)	22	
Periodontal status	Probing depth <4 mm	18 (39%)	17 (37%)	35	1
	Probing depth ≥4 mm	5 (11%)	6 (13%)	11	
Bleeding on probing*		3.74 ± 2.20	3.91 ± 1.88	3.83 ± 2.03	0.77
Age (years)		17.22 ± 1.41	17.78 ± 1.38	17.50 ± 1.41	0.17
Neck circumference (cm)		37.57 ± 2.84	37.87 ± 4.34	37.72 ± 3.63	0.78
Waist circumference (cm)		82.26 ± 11.82	80.78 ± 13.38	81.52 ± 12.51	0.69
Average systolic pressure (mmHg)		110.28 ± 13.36	109.22 ± 10.95	109.75 ± 12.09	0.76
Average diastolic pressure (mmHg)		74.85 ± 8.65	70.00 ± 8.02	72.42 ± 8.61	0.05
Number of decayed, missing, filled teeth**		6.35 ± 4.56	7.52 ± 4.97	6.93 ± 4.75	0.4

Data are shown as mean ± standard deviation (SD) for continuous variables and as counts (percentages) for categorical variables. *p*-values were calculated using two-sided t-tests for continuous variables and chi-square tests for categorical variables. *Average number of teeth based with bleeding on probing on the 6 Ramfjord index teeth. **The DMFT (Decayed, Missing, Filled Teeth) index is a clinical measurement used to quantify dental caries experience by counting the total number of decayed, missing due to decay, and filled teeth. Higher DMFT scores reflect poorer oral health status.

### Distinct diversity profiles in saliva and plaque microbiomes regardless of T1D status

**Figure 1:**
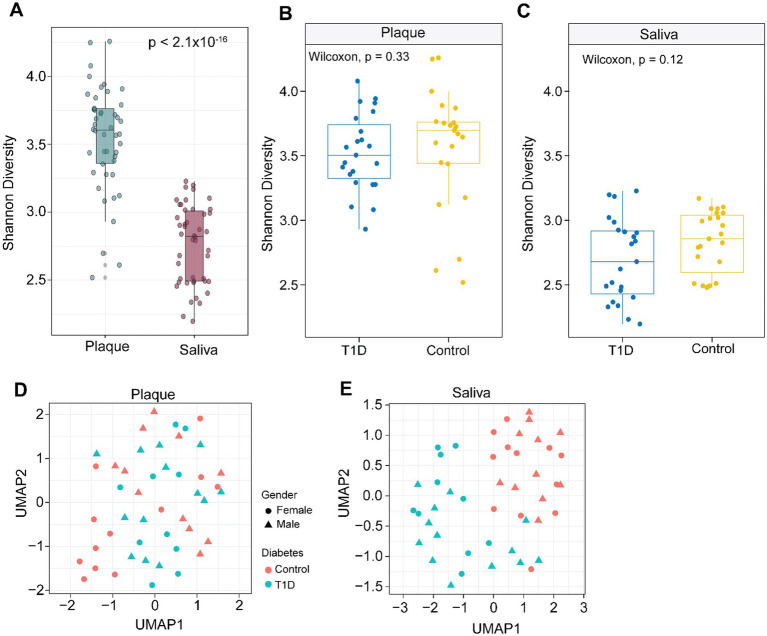
Distinct microbial diversity and composition in saliva and plaque samples in children with and without Type 1 Diabetes (T1D). **(A)** Boxplot of Shannon diversity indices indicates significantly higher alpha diversity in plaque compared to saliva (*p* < 2.2 × 10^−14^, *t*-test). **(B)** Shannon diversity in plaque samples stratified by T1D status shows no significant difference between T1D and control groups (*p* = 0.33). **(C)** Shannon diversity in saliva samples stratified by T1D status also shows no significant difference (*p* = 0.12). **(D)** UMAP plot visualizing normalized microbiome profiles in saliva samples, stratified by diabetes status (salmon color: control, cyan color: T1D) and gender (circle: female, triangle: male), showing a distinct clustering of T1D samples. **(E)** UMAP plot for plaque samples reveals less distinct separation between T1D and control groups compared to saliva.

We compared microbial diversity and community composition between plaque and saliva samples in children with and without T1D (Figure 1). Overall, plaque samples exhibited significantly higher alpha diversity than saliva, as measured by the Shannon diversity index (Wilcoxon *p* < 2.1 × 10^−16^). In contrast, saliva samples show lower diversity with greater variability (Figure 1A). When stratified by disease status, no statistically significant differences in alpha diversity were observed between T1D and control groups within either plaque (*p* = 0.33; Figure 1B) or saliva (*p* = 0.12; Figure 1C), although a slight reduction in salivary diversity was noted in the T1D group.

UMAP analysis suggests that diabetes status is more closely associated with variation in the saliva microbiome than in the plaque microbiome (Figures 1D,E). Plaque samples show no clear clustering by diabetes or gender, whereas saliva samples display distinct separation by diabetes status (Figures 1D,E). UMAP analysis of species-level microbiome profiles revealed minimal separation between T1D and control groups in plaque samples (Figure 1D), indicating limited compositional differences. In contrast, saliva samples demonstrated a more distinct clustering pattern based on T1D status (Figure 1E), suggesting that the salivary microbiome better distinguishes disease-associated microbial shifts. Gender did not influence the clustering patterns in either sample type.

### Distinct phylum-level microbial profiles and abundance patterns between saliva and plaque samples

**Figure 2:**
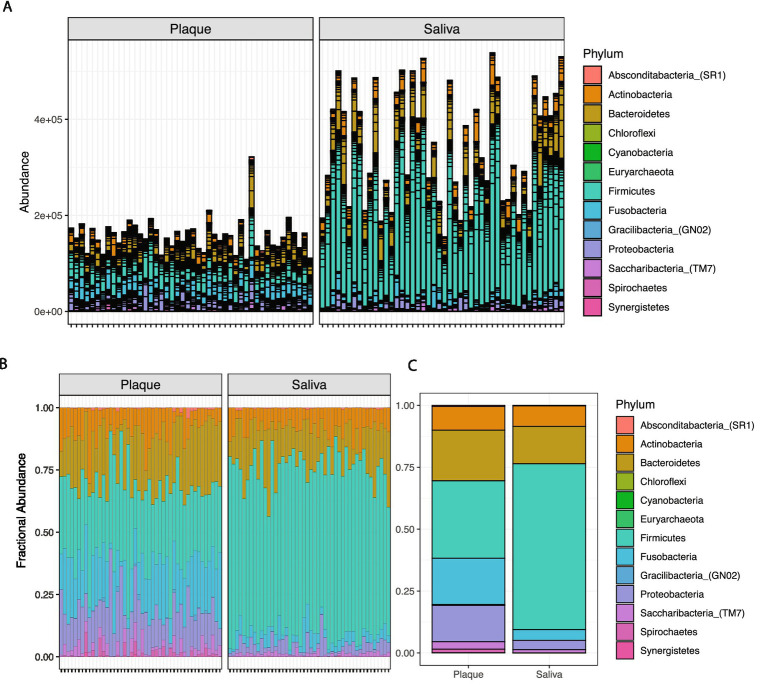
Phylum-level taxonomic composition of oral microbiota in plaque and saliva samples: **(A)** Stacked bar plots displaying absolute abundance of bacterial phyla across individual plaque and saliva samples. Saliva samples exhibit higher overall microbial abundance and greater variability in dominant phyla compared to plaque. **(B)** Relative abundance profiles of bacterial phyla in individual plaque and saliva samples. **(C)** Aggregated bar plot displaying relative abundance of each phylum in plaque and saliva samples.

To compare the taxonomic composition of the oral microbiome between plaque and saliva, we analyzed the abundance of bacterial phyla across all samples. Absolute abundance profiles revealed markedly higher microbial loads in saliva compared to plaque, with consistently greater phylum-level counts observed in most salivary samples (Figure 2A). Saliva exhibited pronounced inter-individual variability in microbial abundance, particularly in dominant phyla such as Bacteroidetes and Firmicutes.

Relative abundance profiles provided further insights into compositional differences between the two oral niches (Figure 2B). In both plaque and saliva, Firmicutes represented the dominant phylum, followed by Bacteroidetes. However, saliva samples showed relatively higher proportions of Firmicutes and Bacteriodetes, while plaque samples had a greater representation of Firmicutes, Proteobacteria, Fusobacteria, and Bacteriodetes. The presence of Absconditabacteria (SR1), Chloroflexi, Cyanobacteria, Euryarchaeota, Gracilibacteria (GN02), TM7 (Saccharibacteria), Spirochaetes, and Synergistetes was noted in both niches but with minor variation in abundance.

Aggregated bar plots summarizing average phylum-level composition (Figure 2C) reinforced these patterns, highlighting saliva’s enrichment in Firmicutes and Bacteroidetes, and plaque’s relative enrichment in Firmicutes, Fusobacteria, Bacteroidetes, and Proteobacteria. These findings demonstrate that while saliva and plaque share core microbial constituents at the phylum level, their overall community structures and microbial loads are distinct, reflecting the niche-specific ecological dynamics of the oral cavity.

### Random forest models reveal distinct salivary microbial signatures predictive of type 1 diabetes

**Figure 3:**
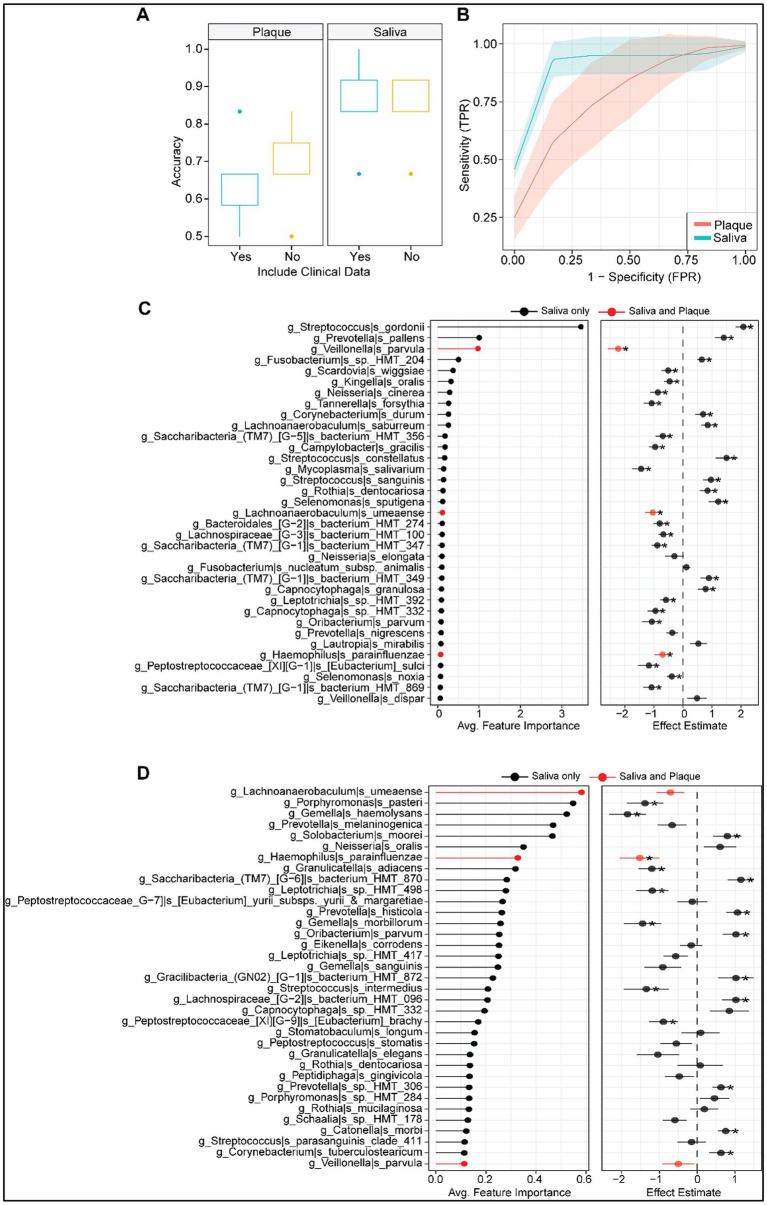
Machine learning performance and microbial feature selection in classifying T1D status using salivary and plaque microbiomes. **(A)** Boxplots of classification accuracy using random forest models for plaque and saliva samples, with and without inclusion of clinical metadata. Saliva-based models outperformed plaque-based models, and inclusion of clinical data did not significantly improve accuracy. **(B)** Receiver operating characteristic (ROC) curves comparing the predictive performance of plaque (red) and saliva (blue) models. Saliva models showed higher sensitivity and specificity with tighter confidence intervals, indicating superior discriminative power. **(C)** Top microbial taxa contributing to classification performance in saliva samples, ranked by average feature importance (left panel). Right panel shows corresponding effect estimates based on a linear model for each taxon; taxa shared between saliva and plaque are highlighted in red. *Significant coefficient from linear model. **(D)** Top microbial taxa driving classification in plaque samples, with average feature importance (left) and effect size estimates (right). Shared taxa between plaque and saliva are highlighted in red. *Significant coefficient from linear model.

To assess the ability of oral microbiome profiles to classify T1D status, we trained random forest machine learning classifiers using species-level taxonomic data from plaque and saliva samples, with and without the inclusion of clinical metadata. Classification models based on salivary microbiome data consistently outperformed those based on plaque data (Figure 3A).

Saliva-based models demonstrated consistently high classification performance, with a median accuracy of approximately (~94%) regardless of whether clinical metadata were included (Figure 3A). In contrast, plaque-based models showed lower predictive accuracy, with a median around (~73%), and exhibited greater variability across model iterations. Notably, the inclusion of clinical metadata did not improve model performance for either biospecimen type, underscoring the strong predictive power of oral microbiome profiles alone.

Receiver operating characteristic (ROC) analysis further confirmed the superior performance of saliva-based models (Figure 3B). The saliva models achieved an average AUC of approximately (~0.94%), indicating excellent discriminative ability with narrow confidence intervals. By comparison, plaque-based models yielded a lower average AUC of around (~0.75%), with broader confidence bands, suggesting less stable and less accurate classification of T1D status.

In saliva samples, models that incorporated clinical metadata achieved an average specificity of 91.7% and an average sensitivity of 96.7%, the same as compared to an average specificity of 91.7% and an average sensitivity of 96.7% when clinical data was excluded (Table 2). In contrast, plaque models showed a slight increase in specificity and slight decline in sensitivity. Plaque models that incorporated clinical metadata achieved an average specificity of 73.3% and an average sensitivity of 73.3%, in which plaque models without clinical metadata achieved an average specificity of 75% and an average sensitivity of 71.7% (Table 2).

**Table 2:** Classification performance of random forest models predicting T1D status using saliva and plaque microbiome data.

Model	Accuracy	Sensitivity	Specificity
Plaque microbiome + clinical data	73.3%	73.3%	73.3%
Plaque microbiome only	73.3%	71.7%	75%
Saliva microbiome + clinical data	94.2%	96.7%	91.7%
Saliva microbiome only	94.2%	96.7%	91.7%

Performance metrics (accuracy, sensitivity, and specificity) are reported for models trained on species-level microbiome profiles from saliva and plaque samples, with and without the inclusion of clinical metadata.

Feature importance analysis identified key microbial taxa contributing to T1D classification in each niche. In saliva (Figure 3C), top discriminatory taxa included *Streptococcus gordonii*, *Prevotella pallens*, *Veillonella parvula*, and *Fusobacterium* sp. *HMT_204*, among others. Several of these taxa, such as *Veillonella parvula* and *Lachnoanaerobaculum umeaense*, were shared between both saliva and plaque (highlighted in red), while others were unique to the salivary niche.

In plaque samples (Figure 3D), taxa such as *Lachnoanaerobaculum umeaense*, *Porphyromonas pasteri*, *Prevotella melaninogenica*, and *Solobacterium moorei* were identified as top predictors. While some overlap was observed between niches, most predictive features were sample-type specific. Effect size estimates were generally more pronounced and consistent in saliva, further supporting its value as a biospecimen for T1D-related microbiome classification.

**Figure 4:**
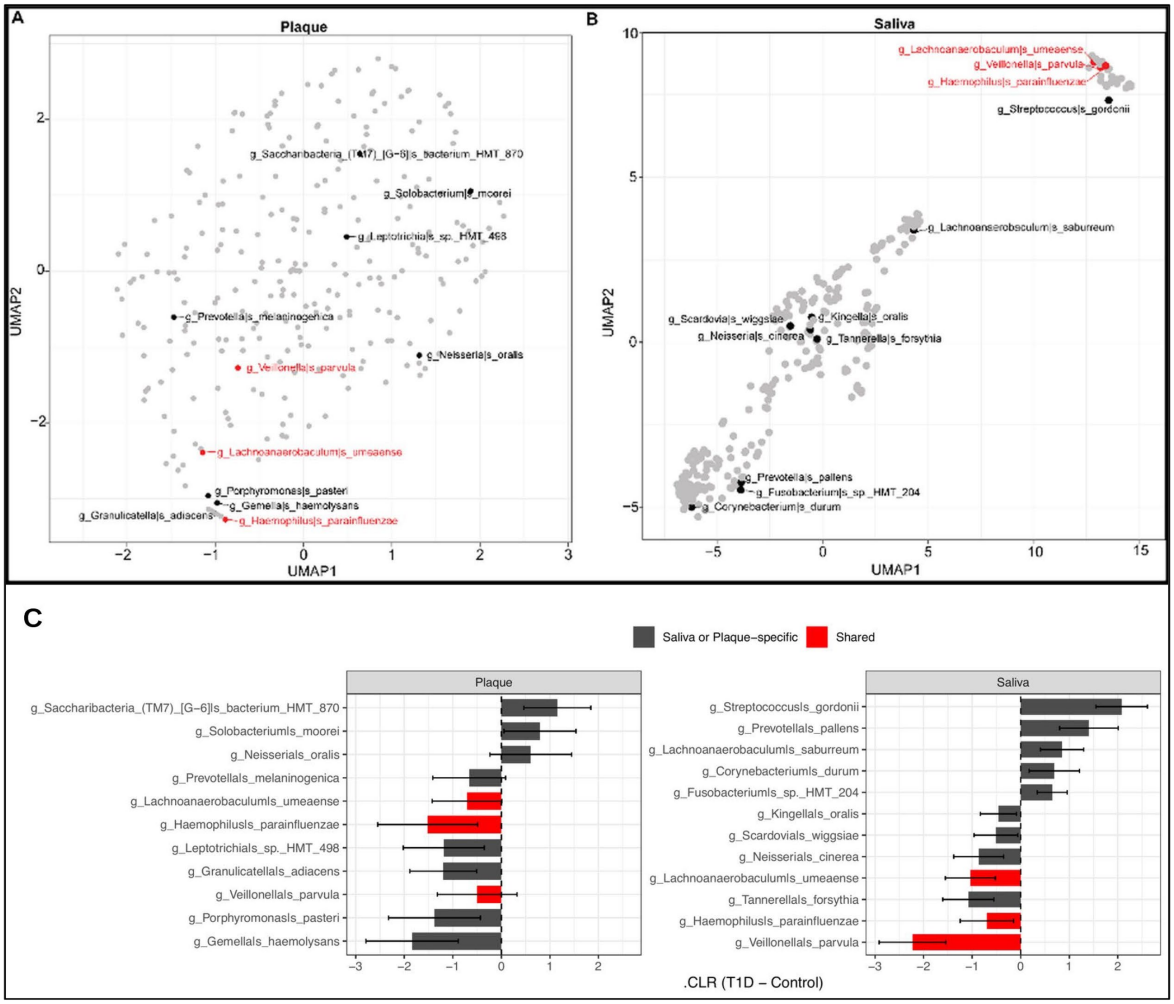
UMAP visualization of microbial taxa differentiating T1D status in plaque and saliva samples: **(A)** UMAP projection of microbial taxa in plaque samples. The top predictive species shared between saliva and plaque in T1D samples are shown in red, including *Veillonella parvula*, *Lachnoanaerobaculum umeaense*, and *Haemophilus parainfluenzae*. Other predictive features are labeled in black, such as *Prevotella melaninogenica*, *Leptotrichia* sp. HMT_498, and *Solobacterium moorei*. **(B)** UMAP projection of microbial taxa in saliva samples. The top predictive species shared between saliva and plaque are shown in red, while other predictive features are shown in black, including *Streptococcus gordonii*, *Kingella oralis*, and *Corynebacterium durum*. The UMAP layout demonstrates clearer spatial segregation of key microbial features in the salivary microbiome compared to plaque. **(C)** Bar plot summarizing the direction and magnitude of change in centered log-ratio (CLR)–transformed abundance for the key microbial taxa highlighted in panels **(A,B)**. Bars represent the difference in abundance between T1D and control subjects (ΔCLR = T1D − Control), with positive and negative values indicating taxa increased or decreased in T1D, respectively. Error bars denote 95% confidence intervals. Taxa shared between saliva and plaque are highlighted in red.

Table 3. summarizes the top 10 salivary microbial taxa ranked by feature importance in predicting T1D status (as shown in Figure 3). For each taxon, the direction of abundance change in children with T1D is indicated, along with literature-based characterizations supporting their potential pathogenic, commensal, or protective roles in the oral environment. We further performed UMAP-based dimensionality reduction of species-level abundance data from plaque and saliva samples (Figure 4) to examine structure among predictive taxa. Shared top predictive species are highlighted in red; other predictive species are shown in black. Plaque embeddings (Figure 4A) exhibit diffuse, weakly defined clusters, with no clear grouping of the shared top predictors. In contrast, saliva embeddings (Figure 4B) show more pronounced structure, including a compact cluster composed of the shared top predictors. Non-shared predictors are distributed broadly across the embedding in both sample types. Figure 4C illustrates the direction and magnitude of abundance changes (ΔCLR) for the top predictive taxa in plaque and saliva samples. The shared taxa (highlighted in red) demonstrated consistent directional changes across both oral niches, suggesting that certain microbial shifts associated with T1D are detectable regardless of sample type.

**Table 3:** Directionality of abundance and literature-based characterization of the top 10 salivary bacteria associated with T1D.

Oral microbiome taxa	Abundance in T1D	Literature-based characterization
*Streptococcus gordonii*	Increased	Functions as an early oral colonizer ordinarily associated with health-compatible niches; however, when host defenses are compromised it can shift towards an opportunistic pathogenic phenotype and is frequently enriched within inflamed niches (Bloch et al., 2024; Kerrigan et al., 2007; Solanki et al., 2024).
*Prevotella pallens*	Increased	Associated with proinflammatory microenvironments, showing heightened relative abundance in dysbiotic biofilms. Evidence indicates that it modulates host cytokine signaling by attenuating IL-6 and IL-8 expression under specific conditions, contributing to immune activation and mucosal disruption (Fteita et al., 2018; Könönen et al., 2022; Könönen and Gursoy, 2022; Xie et al., 2024).
*Veillonella parvula*	Decreased	Promotes mucosal immune tolerance, and its early depletion has been linked to the onset of autoimmunity. Acts as a lactate-utilizing buffer, converting streptococcal lactic acid into weaker acids and simultaneously reducing nitrate to nitrite, which selectively inhibits cariogenic *Streptococcus mutans*. This metabolism elevates salivary pH, protecting enamel from demineralization, and counteracts the hyperglycemic salivary environment (Räisänen et al., 2023; Sangha et al., 2024; Wicaksono et al., 2020; Zhou et al., 2021).
*Kingella oralis*	Decreased	Regarded as a low-virulence commensal that coexists with other early colonizers in health-associated biofilms. It is rarely implicated in pathology and is generally viewed as ecologically neutral or mildly beneficial in sustaining oral homeostasis (Chen, 1996; Dewhirst et al., 1993; Giacomini et al., 2025; Jitvaropas et al., 2022).
*Scardovia wiggsiae*	Decreased	Often an acidogenic species that is linked to early-childhood caries. In our dataset, however, its effect estimate was slightly lower in children with T1D compared to the control group. A plausible explanation is the restriction of low sugar intake in pediatric T1D dietary management, which deprives *S. wiggsiae* of the fermentable substrates that drive its cariogenic activity (Kameda et al., 2020; Kressirer et al., 2017; Tanner et al., 2011).
Fusobacterium sp. HMT 204	Increased	Acts as a “bridge” whose adhesins link early streptococci to late anaerobes. Hyperglycemia up-regulates its invasion genes and triggers greater IL-6 release from host cells, amplifying local inflammation. This glucose-driven virulence, coupled with its bridging role, explains the mildly harmful effect observed in our model (Chang et al., 2023; Fan et al., 2023; Nomura et al., 2024).
*Tannerella forsythia*	Decreased	Forsythia is generally considered a member of the healthy oral microbiome, though its abundance often increases in adults with periodontal disease (Dewhirst et al., 2010). In our pediatric cohort, however, Table 1 shows no statistically significant difference in probing depth between T1D and control groups. This may have obscured any relationship between Forsythia abundance and periodontal status in our sample.
*Corynebacterium durum*	Increased	Opportunistic under prolonged hyperemic stress and pathogenic once anatomical barriers & immunity fail in frail, immunocompromised, or inflamed hosts. This challenges prior research that this bacterium is a commensal associated solely with microbial stability and oral health (Li and Tang, 2025; Redanz et al., 2021; Riegel et al., 1997).
Lachnoanaerobaculum saburreum	Increased	Acid-tolerant, sugar-fermenting species expands in carbohydrate-rich, low-pH niches and is consistently linked to caries and oral dysbiosis. Although present at low levels in healthy niches, its abundance and pathogenic potential rise when epithelial or immune defenses falter (Abola et al., 2023; Mashimo et al., 1981).
*Neisseria cinerea*	Decreased	A health-associated commensal that contributes to microbial diversity and may help suppress the colonization of pathogenic species through competitive exclusion and oxidative metabolism. It is rarely implicated in disease and is more abundant in oral health (Donati et al., 2016; Knapp and Hook 3rd, 1988).

## Discussion

This study compared oral microbiome profiles from two distinct sites, saliva and dental plaque, to determine which more accurately classified children with T1D versus healthy controls. Our findings show that the salivary microbiome demonstrated superior classification accuracy compared to the plaque microbiome in distinguishing T1D status. To the best of our knowledge, no prior research has directly compared saliva and plaque in the context of T1D. Our findings align with previous studies highlight the diagnostic sensitivity of saliva for characterizing systemic conditions.

Research comparing salivary and plaque microbiomes suggests that saliva is associated more with systemic health and inflammatory status, while plaque is better suited for identifying localized oral diseases (Jung et al., 2024; Zhang et al., 2019). For example, salivary microbiome profiles have been shown to outperform plaque in distinguishing between HIV-positive and HIV-negative individuals and are more strongly correlated with systemic inflammatory markers such as the neutrophil-to-lymphocyte ratio, an indicator of chronic inflammatory conditions (Kim et al., 2022; Li et al., 2014; Perez Rosero et al., 2021). These findings suggest that saliva may be more sensitive for testing oral microbiome and its association with systemic immunosuppression (Acharya et al., 2017). Evidence also shows that oral bacteria, particularly from saliva, can translocate into the bloodstream and contribute to systemic inflammation (Kim et al., 2023). In addition, because saliva continuously bathes the oral mucosa, carries microbial signals to the gastrointestinal tract (Kitamoto et al., 2020), and can reflect certain blood biomarkers (Lim et al., 2023), it serves as a critical conduit for host–microbiome interactions that extend beyond the oral cavity.

In contrast, plaque samples tend to exhibit more stable microbial compositions that are less responsive to systemic disease status (Kistler et al., 2015). Plaque has demonstrated stronger associations with localized conditions such as periodontitis severity (Saito et al., 2021) and dental caries progression (Yang et al., 2021). As a biofilm that adheres to tooth surfaces, dental plaque is shaped by site-specific factors like oral hygiene, diet, and tooth morphology (Benahmed et al., 2021; Marsh, 2006), making it a more reliable marker for oral diseases than for systemic health indicators. Our study reveals that the salivary taxa most influential in classifying T1D status exhibit varying patterns of abundance. Several taxa enriched in individuals with T1D, such as *Streptococcus gordonii*, *Prevotella pallens*, and Fusobacterium species, have established associations with pro-inflammatory activities (Bloch et al., 2024; Chang et al., 2023; Fan et al., 2023; Fteita et al., 2018; Kerrigan et al., 2007; Könönen et al., 2022; Könönen and Gursoy, 2022; Nomura et al., 2024; Xie et al., 2024). Conversely, taxa more abundant in control samples, including *Veillonella parvula* and *Neisseria cinerea*, are recognized components of a balanced oral microbiome (Chen, 1996; Dewhirst et al., 1993; Donati et al., 2016; Giacomini et al., 2025; Jitvaropas et al., 2022; Knapp and Hook 3rd, 1988; Räisänen et al., 2023; Sangha et al., 2024; Wicaksono et al., 2020; Zhou et al., 2021). These findings align with prior work indicating that several oral taxa contribute to community-level functions rather than exerting fixed pathogenic or health-promoting effects (Kolenbrander et al., 2002; Kolenbrander, 2000). Their functional roles in processes like metabolic cross-feeding and pH balance are contingent upon the surrounding ecological context (Wicaksono et al., 2020; Mashimo et al., 1981). For instance, while often commensal, *Streptococcus gordonii* can shift toward an opportunistic pathogenic phenotype when host defenses are compromised (Bloch et al., 2024; Chang et al., 2023; Fan et al., 2023; Kerrigan et al., 2007) Similarly, the role of Fusobacterium is influenced by host metabolic state, with high glucose levels promoting its invasive potential and amplifying local inflammation (Chang et al., 2023; Fan et al., 2023; Nomura et al., 2024). Other taxa, such as *Corynebacterium durum* and Lachnoanaerobaculum, also demonstrate pathogenic potential under conditions of dysbiosis or in susceptible hosts (Mashimo et al., 1981; Abola et al., 2023; Li and Tang, 2025; Redanz et al., 2021; Riegel et al., 1997). Therefore, the shifts we observe, such as the increased abundance of *S. gordonii* and *P. pallens* in the T1D-associated state, likely reflect a transition toward a dysbiotic community driven by interactions within a susceptible host environment (Könönen et al., 2022; Kreth et al., 2008; Wang and Kuramitsu, 2005). Contrary to the popular notion that salivary microbes can be universally labeled as either “beneficial” or “pathogenic,” accumulating ecological evidence indicates that most oral taxa exist along a functional continuum modulated by their immediate microenvironment (Marsh, 2006). The data summarized in Table 3 illustrate that several organisms traditionally regarded as harmless commensals can adopt virulence-associated phenotypes, and vice versa, when inflammation, frailty, or immunosuppression compromise anatomic or immunologic barriers. These observations reinforce an ecological perspective in which oral health and disease are driven by dynamic shifts in oxygen tension, pH, host immune status, and community interactions, rather than by any inherent “good” or “bad” property of a single species. In other words, a bacterium that supports homeostasis in one context can behave as a pathobiont in another (Fine and Schreiner, 2023). Collectively, our results support the hypothesis that disease-associated alterations in the oral microbiome reflect context-dependent imbalances within the microbial community, rather than the intrinsic pathogenicity of individual taxa (Feng and Weinberg, 2006; Peng et al., 2022; Rosier et al., 2018).

Our analyses revealed that saliva samples harbored greater microbial abundance and less diversity than plaque, with median read counts nearly twice as high. This higher microbial load can be attributed to saliva’s viscosity and broad exposure to endogenous and exogenous microbial sources (Roblegg et al., 2019; Zaura et al., 2009). On the other hand, dental plaque demonstrated greater compositional stability and structural complexity, driven by its biofilm matrix composed of extracellular polymeric substances (EPS). This matrix enables microbial communities to persist across time and individuals, resisting environmental perturbations (Ciofu et al., 2015; Kolenbrander et al., 2002).

Furthermore, we demonstrated that salivary microbiomes clustered more distinctly by T1D status than plaque microbiomes, suggesting greater sensitivity to systemic metabolic and immune changes (de Oliveira et al., 2016; Nagalaxmi and Priyanka, 2011). Disease-associated taxa tended to co-occur in structured ecological networks, forming tight groupings in both niches. In saliva, taxa such as *Veillonella parvula*, *Rothia dentocariosa*, and *Streptococcus gordonii* formed disease-specific clusters, while in plaque, similar patterns were observed with species like *Gemella morbillorum* and *Porphyromonas pasteri*, though with less separation. These co-occurrence patterns highlight the role of niche-specific microbial consortia in characterizing microbiome related to T1D (Kolenbrander, 2000; Faust et al., 2012; Sudhakara et al., 2018).

A key finding of our analysis was that the microbiome-only model demonstrated superior performance in discriminating between T1D and control subjects compared to a model that also included clinical and demographic metadata. This result suggests that, within our cohort, the microbial signatures themselves were more powerful discriminators of T1D status than the available clinical and behavioral markers. Notably, the addition of clinical variables did not enhance classification accuracy, indicating that salivary microbial signatures may serve as robust and independent biomarkers for T1D. This finding challenges the common assumption that clinical context is essential for achieving high performance in predictive modeling (Chowdhury and Turin, 2020).

Emerging evidence consistently supports our findings that oral microbiomes in individuals with T1D differ significantly from healthy controls. These include both qualitative and quantitative differences in the oral microbiota related to glycemic control in T1D (Abuqwider et al., 2023; Pachoński et al., 2021). Additionally, some reported dysbiosis characterized by increased opportunistic pathogens at the onset of T1D, reversible through improved glycemic management (Yuan et al., 2022). These studies suggest a tight interplay between oral microbial profiles and systemic metabolic control, supporting saliva’s utility for disease monitoring.

While the exact mechanistic links between oral microbiota and T1D pathogenesis remains underexplored, analogous insights from gut microbiome research offers valuable clues. Gut microbiota dysbiosis in T1D reduces beneficial short-chain fatty acids and butyrate production, while increasing lipopolysaccharide biosynthesis (Abuqwider et al., 2023; Yuan et al., 2022). These alterations disrupt glucose and lipid metabolism, exacerbating hyperglycemia and chronic inflammation (Abuqwider et al., 2023; Bell et al., 2022; Pachoński et al., 2021; Sadagopan et al., 2023).

Similar metabolic and inflammatory interactions may occur in the oral cavity. Oral microbiota can interact with mucosal immune cells, leading to local dysbiosis and inflammation (Arweiler and Netuschil, 2016; Meghil and Cutler, 2020). This localized inflammation can trigger cytokine release, which may activate systemic immune responses and contribute to broader metabolic dysfunction (Blasco-Baque et al., 2017; Kochumon et al., 2024; Jacob et al., 2024). Additionally, certain oral bacteria are capable of metabolizing glucose (Goodson et al., 2017; Sakanaka et al., 2021), and elevated salivary glucose levels, which are reflective of systemic hyperglycemia, may promote the proliferation of pathogenic species (Brito et al., 2021; Negrini et al., 2021), further exacerbating oral dysbiosis.

This study has several limitations. First, the sample size was modest, which may limit the generalizability of findings and the detection of less prevalent microbial signatures. Second, while our machine learning models incorporated a range of clinical and behavioral metadata, key glycemic control metrics such as HbA1c, time-in-range, and history of diabetic ketoacidosis were not included in the initial study design. Consequently, these variables could not be integrated into the present modeling framework. We recognize the significance of these metrics as potential drivers of oral dysbiosis in T1D. To address this, we plan to conduct a follow-up mechanistic analysis incorporating these glycemic control variables, using the blood samples already collected from this cohort. This future analysis will allow us to specifically investigate the interplay between glycemic control, the oral microbiome, and T1D status.

Finally, the cohort was recruited from a single geographic region, which may limit applicability to other populations with different environmental exposures or genetic backgrounds. Despite these limitations, this study provides novel comparative data on salivary and plaque microbiomes in T1D and highlights the potential of saliva as a non-invasive biospecimen for systemic disease monitoring.

## Conclusion

Our findings reinforce growing evidence that the oral microbiome, particularly from saliva, has strong discriminatory power to distinguish subjects with T1D from healthy controls. Saliva emerged as a superior and non-invasive biospecimen, offering high classification accuracy and robust performance across multiple analyses. These results highlight the potential of salivary microbiome profiling as a broader tool for monitoring systemic health in pediatric populations. Understanding oral microbial signatures in this context may serve as a foundation for developing predictive biomarkers and assessing disease progression or response to treatment in T1D. Future longitudinal studies are needed to validate these associations and explore the mechanistic links between oral microbiota and metabolic regulation.

## Data Availability

The datasets presented in this study can be found in online repositories. The names of the repository/repositories and accession number(s) can be at: https://www.ncbi.nlm.nih.gov/, PRJNA1347426.
